# Genome-wide identification of the peanut PITP gene family and functional verification of *AhSFH8* in resistance to *Aspergillus flavus* infection

**DOI:** 10.1186/s12870-025-07667-4

**Published:** 2025-11-28

**Authors:** Mengjie Cui, Linjie Chen, Zheng Wu, Lei Shi, Xiangru Xu, Meng Zhang, Feiyan Qi, Xiaobo Wang, Jing Xu, Hua Liu, Bingyan Huang, Wenzhao Dong, Suoyi Han, Xinyou Zhang

**Affiliations:** Institute of Crop Molecular Breeding, Henan Academy of Agricultural Sciences/The Shennong Laboratory/Key Laboratory of Oil Crops in Huang-Huai-Hai Plains, Ministry of Agriculture and Rural Affairs/Henan Provincial Key Laboratory for Oil Crop Improvement, Zhengzhou, 450002 China

**Keywords:** Peanut, Phosphatidylinositol transfer protein (PITP), *Aspergillus flavus* infection, *AhSFH8*

## Abstract

Phosphatidylinositol transfer proteins (PITPs) are essential in eukaryotes for transporting phosphatidylinositol and phosphatidylcholine monomers across intracellular membranes, and they play key roles in regulating plant growth, signal transduction, stress responses, and other vital biological processes. *Arachis hypogaea* L., the crop most susceptible to *Aspergillus flavus*, has not been reported its PITP genes in responses to *A. flavus* infection. In this study, we performed a genome-wide identification of the PITP gene family in the peanut cultivar Tifrunner using the latest reference genome, and analyzed their expression patterns in different peanut tissues and at various time points after *A. flavus* inoculation. Furthermore, we cloned a candidate gene, *AhSFH8* (a member of the PITP family’s SFH subfamily, which contains Sec14-Nodulin or NIJ16 domains) from the highly *A. flavus*-resistant peanut material “J11” and validated its function via overexpression in tobacco. A total of 85 peanut PITP genes (*AhPITPs*) were identified, which were classified into three subfamilies and distributed across 20 chromosomes. Segmental duplication was found to be the main driver for the expansion of this family, and gene structure, motif, and conserved domain analyses confirmed their structural conservation. Promoter cis-acting element analysis showed that SFH subfamily members harbored more stress-related elements, suggesting their involvement in stress responses. Expression profiling revealed that three *SFH* genes (*AhSFH8*, *AhSFH9*, *AhSFH10*) were potentially associated with *A. flavus* resistance. Cloning of *AhSFH8* from “J11” showed its target fragment is 1917 bp in length, with premature translation termination at 1836 bp. Transgenic tobacco overexpressing *AhSFH8* exhibited significantly less leaf yellowing and fewer surface spores than wild-type tobacco after *A. flavus* infection, preliminarily confirming that *AhSFH8* enhances *A. flavus* resistance. Based on gene family identification, promoter analysis, expression patterns and transgenic functional validation, we identified three *SFH* genes potentially associated with resistance to *A. flavus* infection. Our findings provide a foundation for understanding the functions of the peanut *PITP* gene family and the mechanism underlying *AhSFH8*-mediated resistance to *A. flavus* infection, while also offering valuable candidate genes for peanut resistance breeding.

**Supplementary Information:**

The online version contains supplementary material available at 10.1186/s12870-025-07667-4.

## Introduction

Peanut (*Arachis hypogaea* L.), a globally significant oilseed and economic crop, is extensively cultivated across the world [1]. Rich in lipids, proteins, and other nutrients, it holds substantial economic and nutritional value [2, 3]. However, peanuts are vulnerable to multiple biotic stresses during growth and storage, with *Aspergillus flavus* infection being one of the critical factors constraining the sustainable development of the peanut industry [4, 5]. Infection by *A. flavus* not only causes rot and deterioration of peanut kernels, leading to significant reductions in yield and quality, but more severely, under favorable environmental conditions, the fungus produces aflatoxins [6]. These secondary metabolites exhibit strong toxicity, carcinogenicity, and teratogenicity [7]. Upon entering the human body via the food chain, they can severely damage organs such as the liver and kidneys, and even induce malignant diseases like cancer, thereby posing a severe threat to food safety and human health [8]. Thus, in-depth exploration of peanut genes associated with resistance to *A. flavus* infection and systematic elucidation of their resistance mechanisms are of profound theoretical and practical importance for breeding *A. flavus*-resistant peanut cultivars, ensuring safe peanut production, and protecting human health.

Phosphatidylinositol transfer proteins (PITPs) constitute a highly conserved protein family ubiquitously present in eukaryotes, primarily tasked with the intracellular trafficking of lipid molecules (e.g., phosphatidylinositol) and involved in the regulation of lipid metabolic processes [9–11]). In plant cells, by participating in lipid transport and metabolism, PITPs play an indispensable role in critical physiological processes, including cellular signal transduction, maintenance and repair of biomembrane structures, and cellular responses to various stresses [12–14]. In recent years, advances in molecular biology research have revealed that PITP family members play a pivotal role in plant responses to biotic and abiotic stresses. Based on the characteristics of conserved domains in plant PITP proteins, they can be categorized into three types: proteins containing only the Sec14 domain (PITP), those with the Sec14-GOLD domain (PATL), and those harboring the Sec14-Nodulin or NIJ16 domain (SFH) [15–18].

Recent studies have demonstrated that PITP genes play a pivotal role in plant responses to stresses. Heterologous overexpression of wheat *TaPITP-7B* in *Arabidopsis thaliana* L. significantly enhanced the salt stress tolerance of transgenic plants. Under salt stress, transgenic plants exhibited higher germination rates, longer primary roots, greater soluble sugar accumulation, and elevated antioxidant enzyme activities compared to wild-type plants, accompanied by lower levels of oxidative damage [19]. Yang et al. [20] employed CRISPR/Cas9-mediated gene editing to silence *AtPITP7* in *Arabidopsis* and *OsPITP6* in rice, revealing reduced phosphorus uptake efficiency in both species, which in turn impaired normal plant growth. Conversely, overexpression of *OsPITP6* increased tiller number and grain yield in rice. Mao et al. [21] observed that upon infection with *Sporisorium scitamineum*, sugarcane (*Saccharum officinarum* L.) resistant cultivar Yacheng 05–179 showed upregulated expression of *ScSFH1*, *ScSFH2*, *ScPATL1*, and *ScPATL2*, whereas susceptible cultivar ROC22 exhibited increased expression of *ScPITP-1* and *ScPITPp*. Additionally, transient overexpression of *ScPITP-1* and *ScPITPp* in tobacco leaves enhanced resistance to the tobacco pathogen *Ralstonia solanacearum*. Moreover, overexpression of maize *ZmPITPp* in *Arabidopsis* significantly enhanced cold stress tolerance [22]. In summary, the PITP gene family exhibits complex and diverse functions in plant development and abiotic stress responses. Through precise regulation of phospholipid metabolism and signal transduction, these genes not only play critical roles in cell division, photosynthesis, and root development but also provide essential molecular support for plant adaptation to adverse environments such as salt, drought, and low temperature. These studies have established a solid foundation for further elucidating PITP molecular mechanisms and their potential applications in crop improvement [12–14].

Researchers have demonstrated that genetic engineering technologies hold great promise for enhancing stress resistance in peanuts. Yu et al. [23] found that overexpression of the *AhAftr1* gene in peanuts significantly reduced aflatoxin levels in kernels. To date, PITP has been characterized in various crops including *Arabidopsis thaliana*, tobacco, soybean, maize, wheat, cotton, sugar beet, and sugarcane, where it plays crucial roles in biological processes such as biotic and abiotic stress signal transduction, morphogenesis, and root hair development [22, 24, 25]. However, systematic identification of the PITP gene family in peanuts and research on its functions in resisting *Aspergillus flavus* infection remain unexplored. These knowledge gaps severely hinder in-depth understanding of the molecular mechanisms governing peanut resistance to *A. flavus* infection [21, 24]. The present study conducted genome-wide systematic analysis to identify all members of the PITP gene family in the cultivated peanut Tifrunner. Comprehensive analyses of their sequence features, chromosomal distribution, phylogenetic relationships, and gene duplication patterns were performed to elucidate the evolutionary dynamics of this gene family. Additionally, combined with promoter cis-acting element analysis and transcriptome data, candidate genes potentially involved in peanut resistance to *A. flavus* infection were screened. The key candidate gene *AhSFH8* were further cloned, subjected to sequence analysis, and functionally validated to clarify their specific roles in the process of peanut resistance to *A. flavus* infection. This study aims to provide a robust theoretical foundation for in-depth exploration of the biological functions of the peanut PITP gene family and the molecular mechanisms of peanut resistance to *A. flavus* infection, while also offering valuable candidate gene resources for molecular breeding of *A. flavus*-resistant peanuts.

## Meterials and methods

### Experimental materials

The experimental materials comprised two peanut germplasms: “J11”, a high-resistance line against *A. flavus* infection [26, 27], introduced from the International Crops Research Institute for the Semi-Arid Tropics (India); and “Zhonghua 12”, a high-susceptibility line to *A. flavus* [27], obtained from the Oil Crops Research Institute of the Chinese Academy of Agricultural Sciences. Both materials were provided by the Peanut Research Team of Henan Institute of Crop Molecular Breeding and planted in May 2023 under standard field conditions, with the following specifications: six rows per germplasm, 40 plants per row, 40 cm row spacing, 50 cm ridge spacing, 15 cm hole spacing, and two seeds per hole. Upon reaching the maturity stage, peanut pods were harvested, placed in harvest bags, and air-dried until their moisture content fell within the safe storage range (moisture content < 10%). Following this air-drying step, the pods were stored in a warehouse. Subsequently, the pods were shelled, and the resulting kernels were placed in a seed storage box (maintained at 4℃ with 10% relative humidity) for use in subsequent research.

The tobacco variety “*Nicotiana benthamiana*” K326, used for transgenic assay, was purchased from Wuhan Boyuan Biotechnology Co., Ltd. The *A. flavus* strain As 3.4408, characterized by strong infectivity and high toxin-producing capacity, was provided by the Key Laboratory of Agricultural Biological Toxin Detection, Ministry of Agriculture, affiliated with the Oil Crops Research Institute of the Chinese Academy of Agricultural Sciences. Escherichia coli competent cells DH5α and *Agrobacterium tumefaciens* strain GV3101 were sourced from Beijing Tsingke Biotechnology Co., Ltd. Additionally, the overexpression vector pBWA (V) HS was acquired from Wuhan Boyuan Biotechnology Co., Ltd.

### Identification of the peanut PITP gene family

Genomic data for the cultivated peanut (*A. hypogaea* Tifrunner) [28] and diploid wild relatives (*Arachis duranensis* and *Arachis ipaensis*) [29] were downloaded from the Peanutbase database (https://www.peanutbase.org/). Genomic information for *Arabidopsis thaliana* L., *Medicago truncatula* L., and *Glycine max* L. were retrieved from EnsemblPlants (https://plants.ensembl.org/index.html) [30]. The hidden Markov model file PF13716, corresponding to the PITP gene family, was obtained from the Pfam database (http://pfam.xfam.org/) [31]. The hmmsearch tool within HMMER3.1 software was employed to perform alignment against the total protein database of cultivated peanut Tifrunner, with a threshold set at E < 1e^− 5^ [32]. Concurrently, a local BLAST search was conducted against the total protein database of cultivated peanut Tifrunner using 32 previously identified PITP proteins from Arabidopsis as reference sequences. Candidate proteins derived from both methods were combined and subjected to deduplication. The retrieved PITP protein sequences were subsequently submitted to the online InterPro tool (http://www.ebi.ac.uk/interpro/search/sequence-search) [33] and the Pfam database to screen for members containing conserved PITP domains, which were designated as the final candidate genes. Information on PITP gene family members was acquired from the cultivated peanut genome database. The physicochemical properties of the proteins were analyzed using the ExPASy server (https://web.expasy.org/protparam/) [34], and subcellular localization predictions for PITP family genes were performed via Plant-Ploc (http://www.csbio.sjtu.edu.cn/bioinf/plant/) [35].

### Sequence and evolutionary analysis

Multiple sequence alignment was conducted using DNAMAN V6 and SnapGene software (www.snapgene.com) [36]. Chromosomal localization of PITP family genes were generated with Mapchart (v5.4.6) [37], and family members were named according to their chromosomal positions. A phylogenetic tree was constructed via MEGA 11 software employing the Maximum Likelihood Method [38], followed by visualization refinement using the iTOL platform (https://itol.embl.de/) [39]. The MCScanX tool integrated in TBtools was utilized to analyze and visualize intra- and interspecific duplication events of PITP gene family members [40]. Conserved motifs of the PITP gene family were predicted through the MEME server (http://meme-suite.org/tools/meme) [41], with the number of motifs set to 10 and other parameters kept as default. Cis-acting elements within the promoter region (2000 bp upstream of the the coding region of *AhPITPs*) were predicted using PlantCARE (http://bioinformatics.psb.ugent.be/webtools/plantcare/html/) [42]. TBtools was applied to visualize the evolutionary relationships, gene structures, conserved motifs, and promoter cis-acting elements of PITP genes.

### Gene expression pattern analysis

Based on transcriptome data from various peanut tissues publicly available in NCBI (PRJNA291488) [43] and peanut kernels at different time points after *A. flavus* inoculation previously published by our laboratory (PRJNA825125) [27], the Heat Map function in TBtools software was utilized to analyze the tissue-specific expression profiles of *AhPITP* gene family members, as well as their expression differences in resistant versus susceptible materials at various stages post-*A. flavus* inoculation, with heat maps generated accordingly. For the candidate gene *AhSFH8*, RT-qPCR was employed to validate its expression patterns at 0, 1, 2, 3, 5, and 7 days after *A. flavus* infection in peanut kernels. RT-qPCR primers (Table S1) were designed using Primer 5 software. The reaction system and procedure were set up following the instructions provided with the Talent qPCR PreMix kit (SYBR Green, TIAN GEN, Beijing). Each sample included three biological replicates. Experimental data were analyzed using the 2^−ΔΔCT^ method [44].

### Cloning of *AhSFH8* from highly resistant peanut material “J11”

Peanut kernels at 85 days after flowering (DAF) were selected for total RNA extraction using the RNAprep Pure Polysaccharide & Polyphenol Plant Total RNA Isolation Kit (DP 441, TIAN GEN, Beijing). Complementary DNA (cDNA) was synthesized via reverse transcription with the Fasting RT Kit (With gDNase) First-Strand Synthesis Kit (TIAN GEN, Beijing). Specific primers were designed based on the CDS sequences of *AhSFH8* (*Arahy. HTAW4M*) from the Tifrunner genome. The full-length open reading frames (ORFs) of the genes were amplified by PCR, ligated into the pMD19-T vector, and confirmed by sequencing.

### Genetic transformation and phenotypic analysis of tobacco

The *pBWA(V)HS-AhSFH8* overexpression vector was also constructed via homologous recombination. Following transformation of the vector into Agrobacterium, tobacco leaves were inoculated, air-dried, and then placed on co-culture medium for dark incubation for 48 to 72 h. After 2 days of co-culture, the leaves were transferred to induction medium to promote callus formation. Approximately 10 days later, upon callus development, qualified calli were selected and transferred to selective medium, followed by incubation at 23 ± 2℃ for 15 to 30 days. Vigorously growing positive calli after the second round of screening were inoculated onto differentiation medium (4 to 5 calli per dish) and cultured at 23℃ under a 16-h light/8-h dark photoperiod for 15–30 days. Seedlings formed during differentiation were transferred to shoot elongation medium for 7 to 10 days of growth. Genomic DNA was extracted from leaves of transgenic tobacco using the Plant Genomic DNA Kit (DP305-03, TIAN GEN, Beijing). Successful integration of the *AhSFH8* was verified via hygromycin resistance screening and PCR-based positive identification. Using aseptic techniques, uniformly sized *AhSFH8*-overexpressing transgenic tobacco and wild-type controls with robust growth were selected from the growth chamber. Healthy leaves were aseptically excised and trimmed into 4 square segments (2 cm×2 cm each), which were then immersed in a *A. flavus* spore suspension (2 × 10^6^ spores/mL) for 20 min. Treated leaf segments were placed on moist filter paper containing 0.1% Tween-80 and incubated in a 25℃ growth chamber with light for 8 days, during which humidity was maintained by regular spraying of 0.1% Tween-80 solution. Post-incubation, phenotypic differences between the two groups of infected leaves were observed and compared. For quantitative analysis, leaf samples were immersed in 15 mL of 0.1% Tween-80 solution and agitated for 10 min to elute surface spores. The number of *A. flavus* spores on leaf surfaces was counted using a 10× light microscope.

## Results

### Acquisition of AhPITP gene family members and analysis of their physicochemical properties

In this study, the peanut protein database was interrogated using the hidden Markov model file (PF13716). Concurrently, a BLAST search was performed on cultivated peanuts with reference to the protein sequence of an Arabidopsis PITP family member (NP_001328041.1). Candidate proteins identified from both methods were merged and deduplicated, followed by sequence similarity and conserved domain analysis. Ultimately, 85 members of the peanut PITP gene family were identified and named based on the chromosomal locations of members within different subfamilies. Physicochemical property analysis revealed that AhPITP family proteins vary in amino acid length from 140 to 698 residues, with molecular weight ranging from 15.46 to 79.75 kDa, and isoelectric point between 4.75 and 9.93. Prediction of subcellular localization indicated that all 85 peanut PITP family proteins are localized in the cytoplasm (Table S2).

### Analysis of evolutionary relationships and duplication patterns of *AhPITP* family genes

**Fig. 1:**
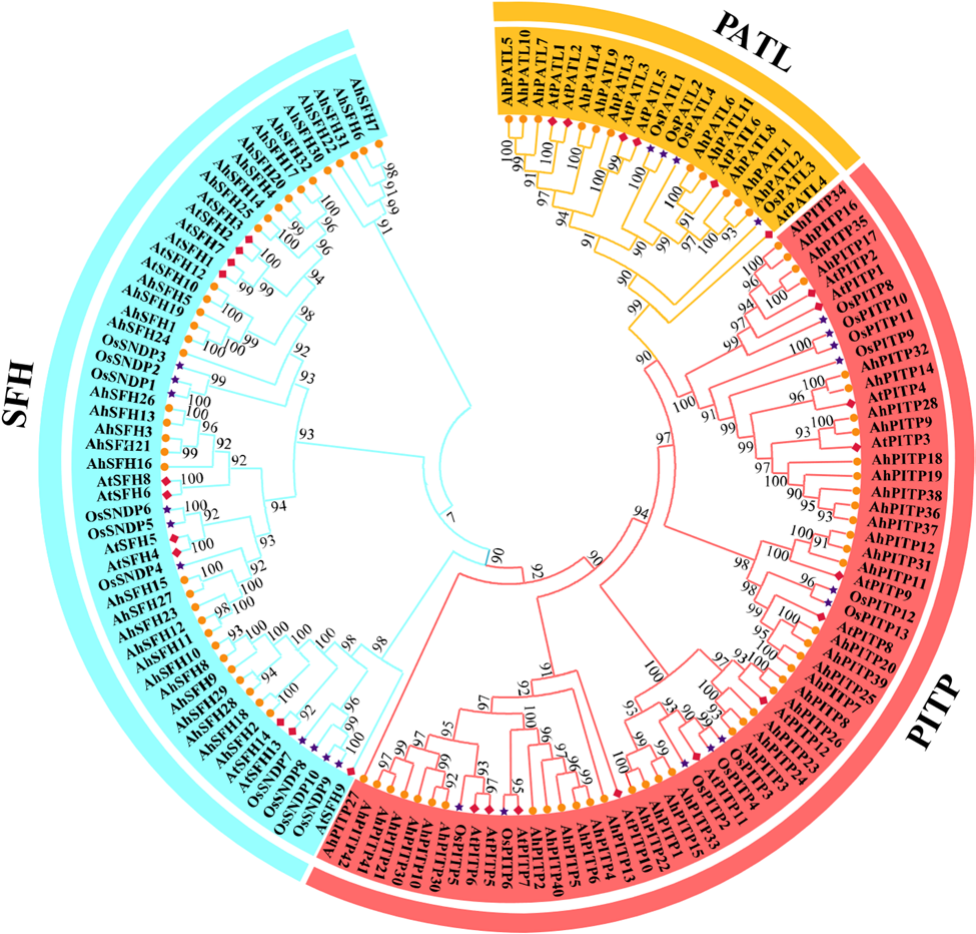
Phylogenetic tree of *PITP* gene family members from *Arachis hypogaea* L., *Arabidopsis thaliana* L., and *Oryza sativa* L

Previous studies have demonstrated that members of the PITP gene family can be categorized into three subfamilies: SFH, PATL, and PITP [25]. To analyze the evolutionary characteristics of the PITP protein family across different species, a phylogenetic tree was built using 85 PITP family members from peanut, 32 from Arabidopsis, and 26 from rice. As depicted in Fig. 1, based on clustering information and protein sequence features, members of the plant PITP gene family were classified into PATL, PITP, and SFH subfamilies, consistent with the established classification. Furthermore, phylogenetic tree was constructed in this study using 85 peanut PITP gene family members. The results (Fig. S1A) revealed that members of the cultivated peanut PITP family could also be classified into three groups, which was largely consistent with the grouping of the plant PITP gene family. Conserved domain analysis revealed that all AhPITP proteins contain a Sec14 conserved domain at their N-terminus. In addition, members of the AhSFH subfamily possess a Nodulin conserved domain at the C-terminus, while those of the AhPATL subfamily contain a GOLD conserved domain at the C-terminus (Fig. S1B, Table S3). This indicates that plant *PITP* genes are highly conserved during evolution. However, variations exist in the length and structure of amino acid sequences among members of different species, implying potential functional diversity. This result further validates the accuracy and reliability of the identification method employed for the peanut PITP gene family in this study.

**Fig. 2:**
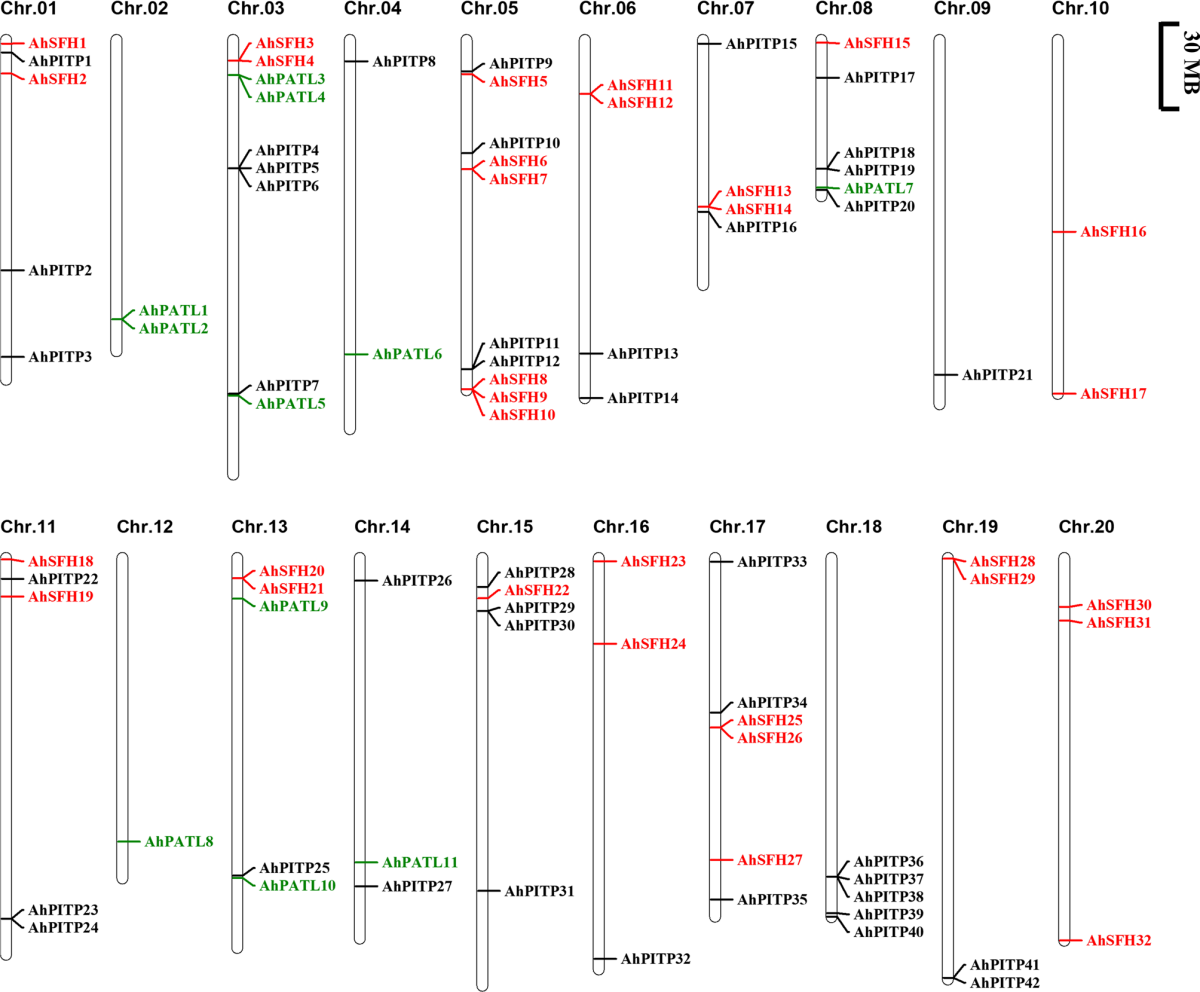
Genome distribution of *AhPITP* on peanut chromosomes

**Fig. 3:**
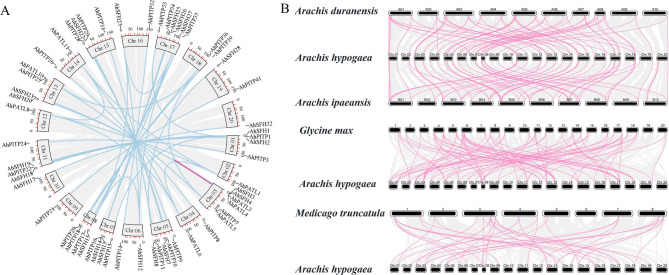
Syntenic relationships. **A**, schematic representation of the inter-chromosomal relationships among *AhPITP* genes. Gray lines indicate syntenic blocks in the *A. hypogaea* L. genome, whereas blue and pink lines indicate duplicated *AhPITP* gene pairs. The chromosome number is indicated above each chromosome. **B**, synteny among *PITP* genes in *A. duranensis*, *A. ipaensis*, *Medicago truncatula* L., *Glycine max* L., and *A. hypogaea* L. genomes

To characterize the chromosomal distribution of *AhPITP* gene family members in peanut, this study performed visual analysis of their physical locations. Results indicated that 85 *AhPITP* genes are randomly distributed across 20 peanut chromosomes. Among these, members of the PITP subfamily exhibit the broadest distribution, spanning 16 chromosomes, followed by SFH subfamily members; PATL subfamily members are restricted to 7 chromosomes (Fig. 2). Gene duplication events are ubiquitous in plant genome evolution, enabling gene amplification through mechanisms such as whole-genome duplication, tandem duplication, and chromosomal segment duplication [45]. To investigate the specific amplification mechanisms of *AhPITP* genes in the peanut genome, detailed intra- and interspecific collinearity analyses were conducted. Comparison of intraspecific collinear regions revealed 41 pairs of segmentally duplicated genes and 1 pair of tandemly duplicated genes within the AhPITP family, suggesting that segmental duplication events may represent a key driver of family expansion (Fig. 3A). Interspecific orthologous relationship analysis identified numerous duplicated gene pairs between cultivated peanut and both alfalfa and soybean (Fig. 3B).

### Analysis of gene structure and conserved motifs of AhPITP family members

**Fig. 4:**
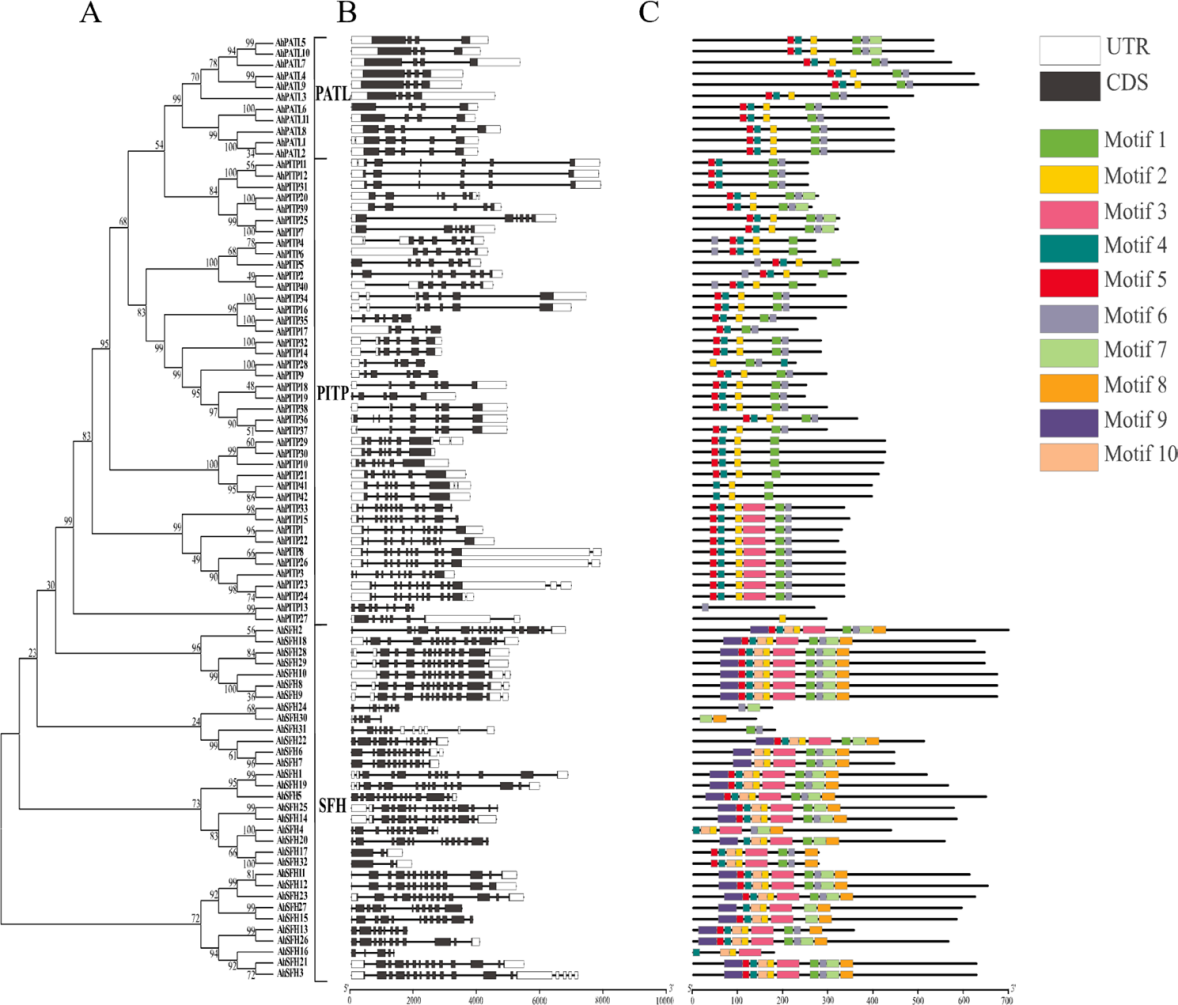
Gene structure and protein conserved motifs analysis of *AhPITPs.* **A**, Phylogenetic tree; **B**, Gene structures; **C**, Protein conserved motif

To further characterize the structural features of the PITP gene family in peanut, this study analyzed the intron/exon architecture and conserved motif profiles of AhPITP family members. Gene structure analysis (Fig. 4B) demonstrated that all AhPITP family members contain both introns and exons. Intron-exon distribution patterns are relatively consistent among members of the same AhPITP subfamily, whereas significant variations in the number and length of introns/exons were observed among different subfamilies. Protein conserved motif analysis (Fig. 4C, Fig. S2) revealed that the conserved domains of AhPITP family proteins are primarily composed of motif 2 and motif 4. The number of conserved PITP motifs is generally consistent within the same subfamily, although some genes exhibit motif gains or losses. For instance, a few PITP subfamily members possess an additional motif 3 compared to other subfamily members; SFH subfamily members such as *SFH16*, *SFH24*, and *SFH30* lack multiple motifs relative to their subfamily counterparts. The AhPITP family also contains unique conserved motifs—specifically motif 8, 9, and 10—which are exclusively present in SFH subfamily members. AhPITP proteins within the same subfamily display similar motif order and counts, whereas motif types and numbers differ across subfamilies. These divergent motifs may contribute to functional differentiation of AhPITP proteins among subfamilies. Overall, comparative analysis of gene structures and conserved motif features revealed a high degree of similarity among members of the same subfamily, indicating that most *AhPITP* genes maintain a high level of structural conservation within their respective subfamilies.

### Analysis of promoter cis-acting elements

**Fig. 5:**
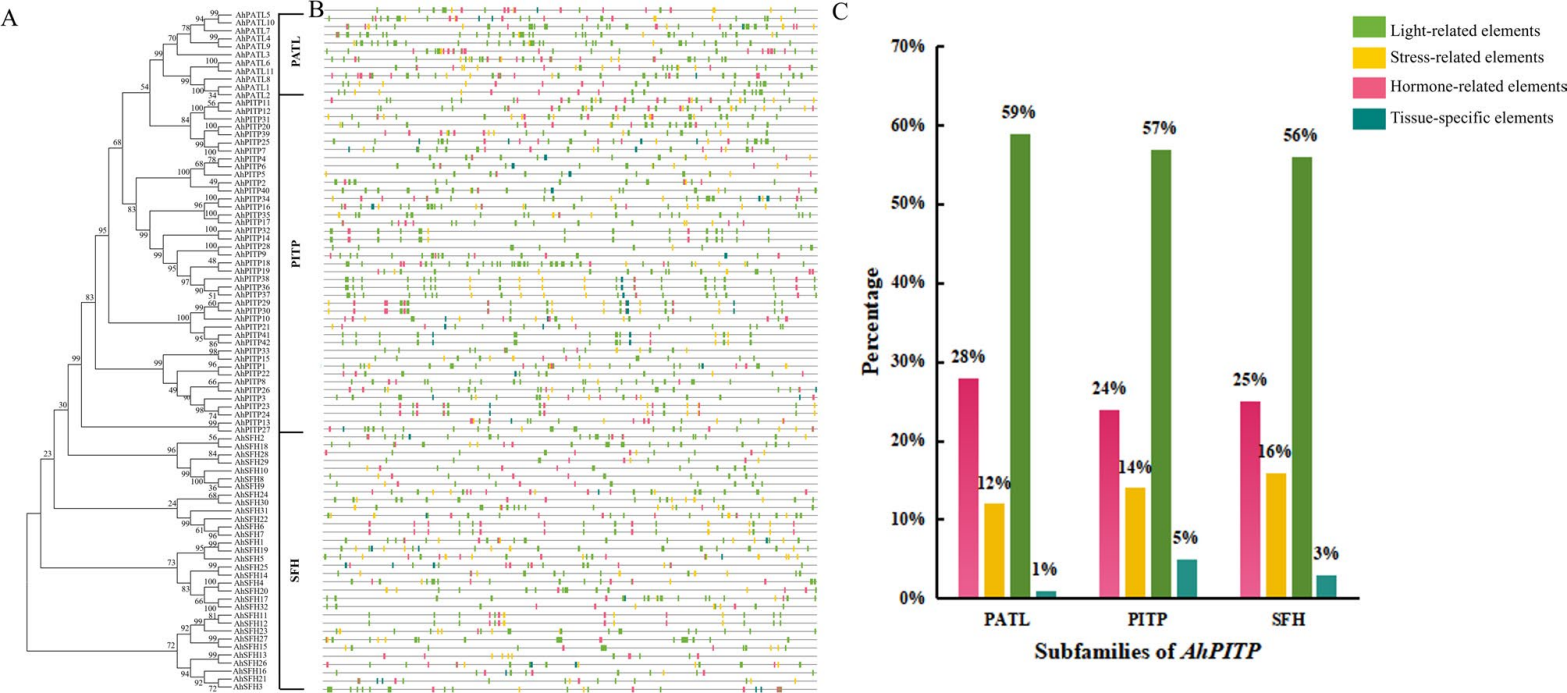
Identification of the cis-acting elements in the 2-kb *AhPITPs* promoter region. **A**, Phylogenetic tree; **B**, *AhPITP* promoter cis-acting elements. Four types of cis-acting elements are differentiated by color. The lengths of sequences can be estimated using the scale at the bottom. **C**, Percentage of hormone- and stress-related elements in different subfamilies

As a key genetic element involved in the regulation of gene expression, the promoter comprises a core promoter region and a regulatory region. In this study, a 2000 bp sequence upstream of the coding region of each *AhPITP* gene was extracted, and the cis-acting regulatory elements within their promoter regions were analyzed. The results (Fig. 5) revealed that the promoter regions of all 85 AhPITP genes contain hormone-related elements (e.g., ABRE) and stress-responsive elements (e.g., TC-rich repeats), suggesting that *AhPITP* genes may play a role in the stress resistance responses of peanut by responding to hormonal and stress signals. Further analysis indicated that there are differences in the number and distribution of cis-acting elements among members of different subfamilies. Notably , the proportion of stress-responsive elements and hormone-responsive elements in SFH subfamily members is the highest at 41%, surpassing that of the PITP and PATL subfamily (Fig. 5C), suggesting that SFH subfamily members may play a more crucial role in peanut resistance to biotic stress.

### FPKM value analysis of peanut PITP family genes in different tissues and at various time points after *A. flavus* inoculation

**Fig. 6:**
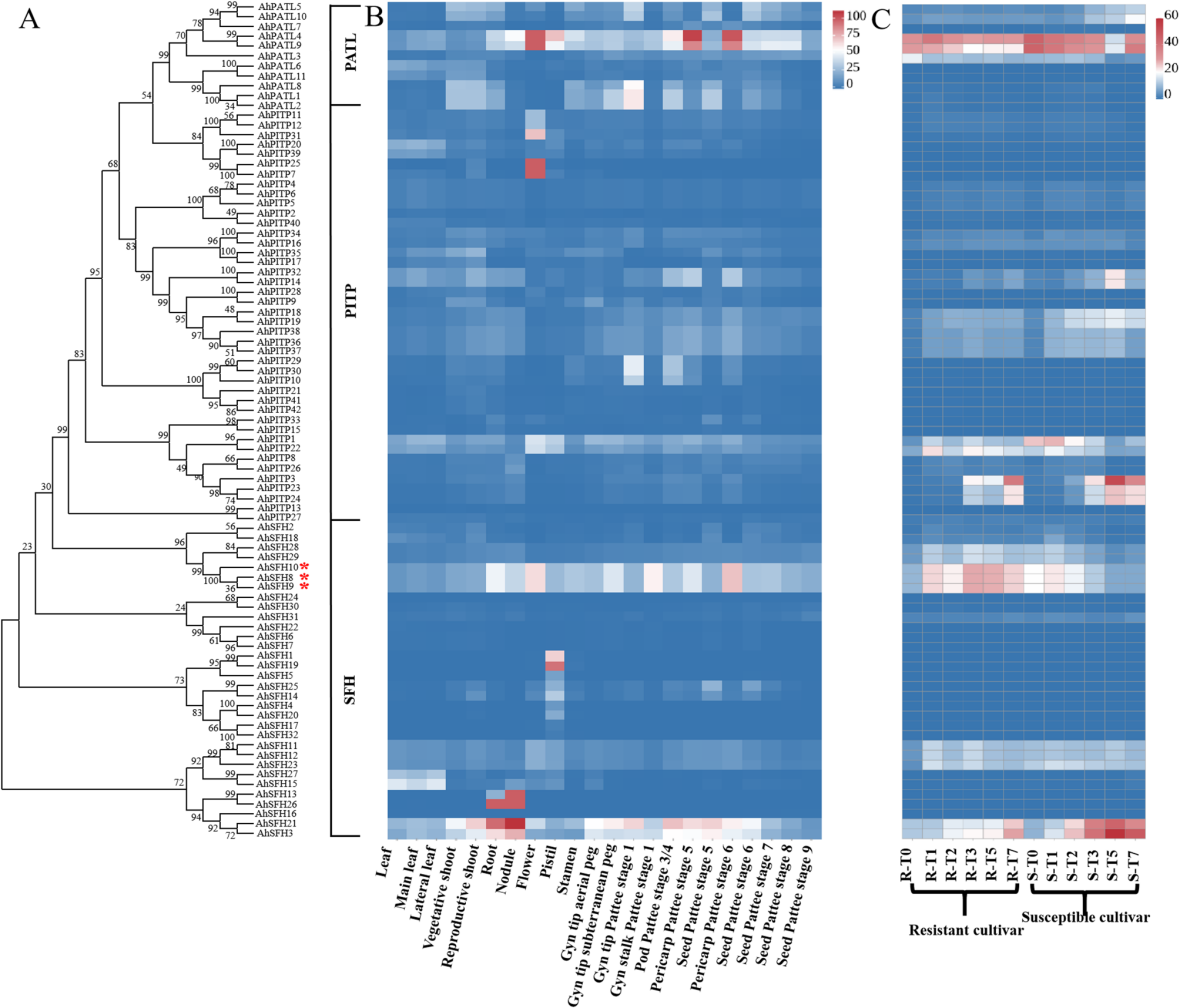
Expression characteristics of *AhPITP* genes. **A**, Phylogenetic tree. Expression patterns of *AhPITP* genes in peanut different tissues (**B**) and in peanut kernels at various time points after inoculation of *A. flavus* (**C**)

In this study, transcriptome data from various peanut tissues publicly available from NCBI [43], as well as previously generated transcriptome datasets of resistant and susceptible peanut lines in response to *A. flavus* infection at different stages [27] were utilized to analyze the tissue-specific expression profiles of *AhPITPs* and their expression patterns at distinct time points post-*A. flavus* inoculation. The results revealed that the expression levels of these family members varied across different peanut tissues, with members of the same subfamily exhibiting similar expression patterns. For instance, most PITP subfamily members were highly expressed in roots, whereas the majority of SFH subfamily members showed high expression levels in flowers and kernels. At the same time point following *A. flavus* infection, the expression levels of certain genes-particularly *AhSFH8*, *AhSFH9* and *AhSFH10* from the SFH subfamily-were significantly higher in resistant materials compared to highly susceptible ones, suggesting their potential role as important positive regulators in the resistance of peanut materials to *A. flavus* infection. Based on these findings, *AhSFH8*, *AhSFH9* and *AhSFH10*, which displayed significantly upregulated expression, were selected as candidate genes associated with peanut kernel resistance to *A. flavus* infection for subsequent functional investigations (Fig. 6).

### Cloning and expression pattern analysis of *AhSFH8*

**Fig. 7:**
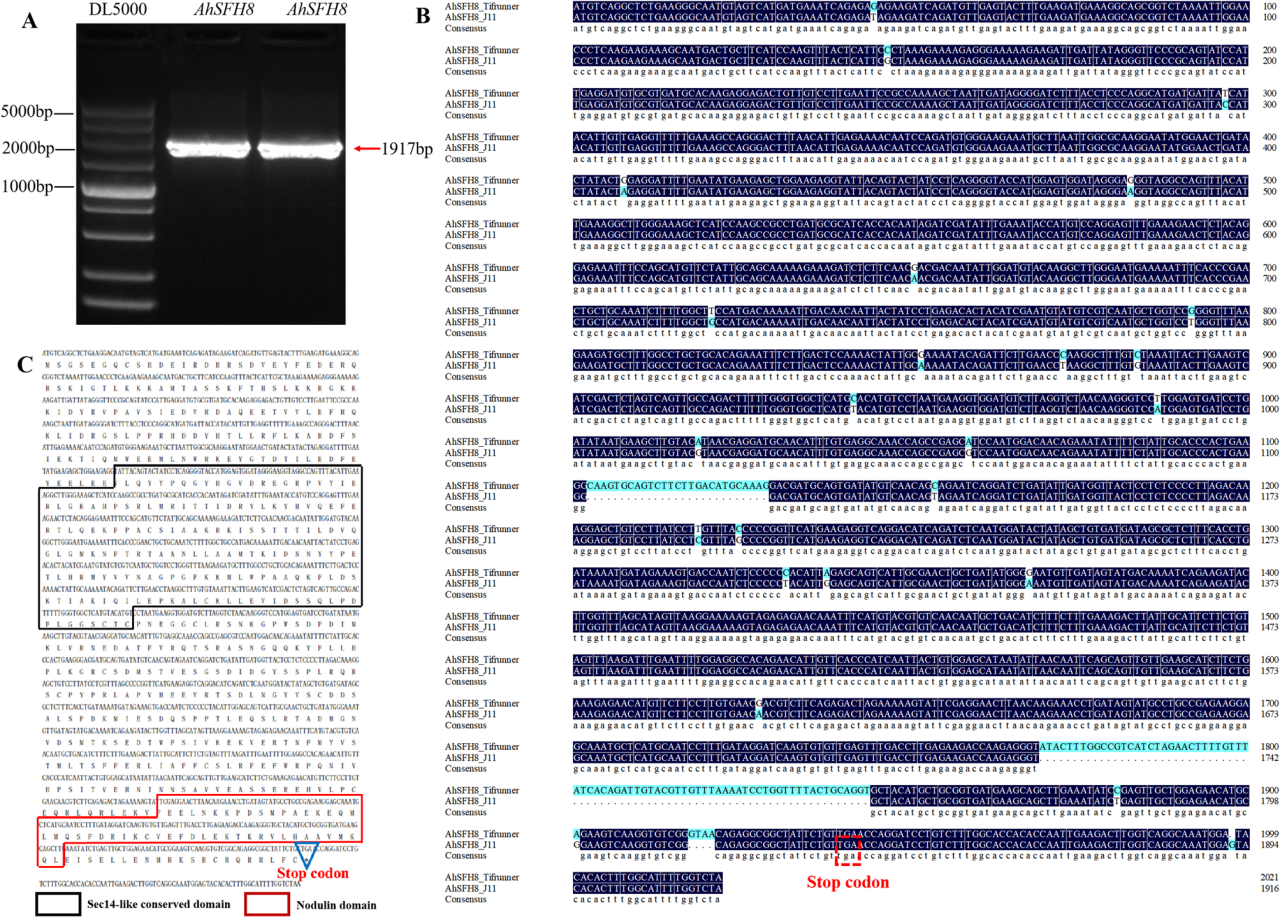
Cloning and characterization of *AhSFH8*. **A**, PCR amplification. **B**, sequence alignment analysis of *AhSFH8* CDS in “J11” and “Tifrunner”. **C**, conserved domain analysis of AhSFH8 protein

**Fig. 8:**
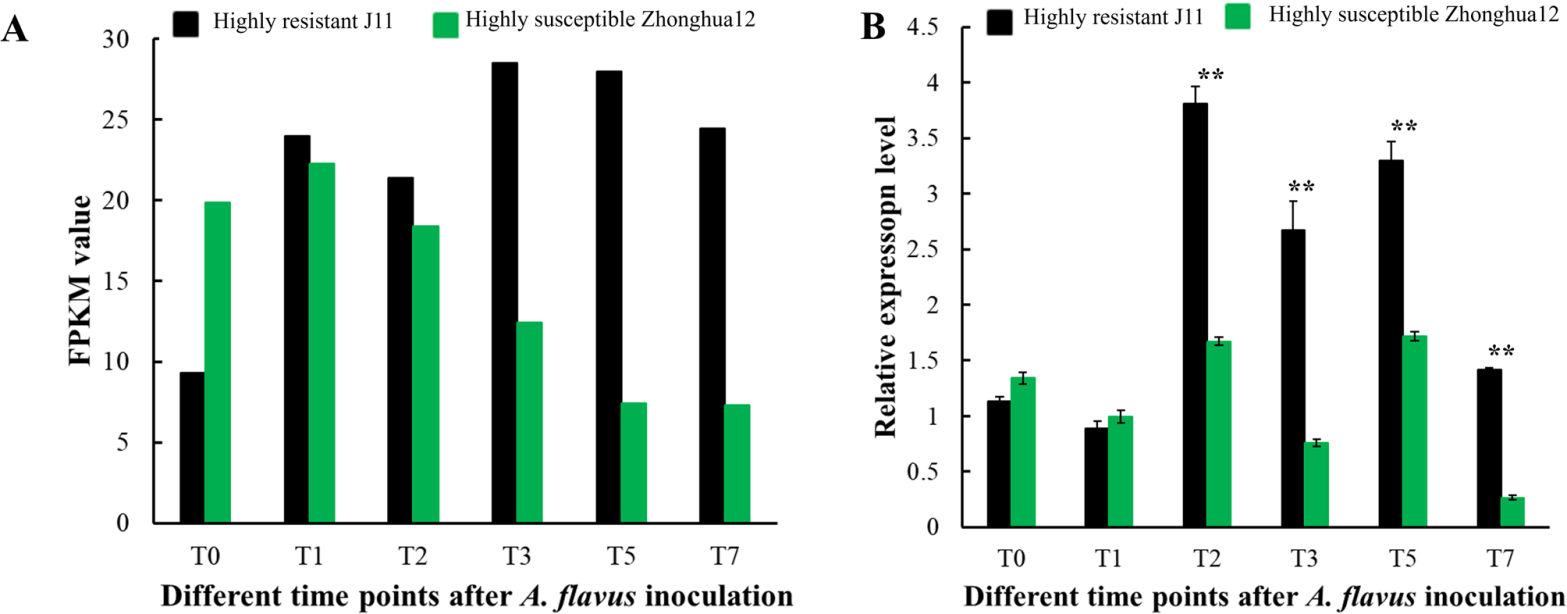
Expression analysis of *AhSFH8* at different times after inoculation of *A. flavus.* **A**, expression analysis based on RNA-seq. **B**, expression analysis based on RT-qPCR. **: significant at *P* ≤ 0.01

According to the genomic annotation of cultivated peanuts, the coding region sequences of *AhSFH8*, *AhSFH9* and *AhSFH10* are completely identical, and the subsequent verification of gene functions will mainly focus on *AhSFH8*. Using cDNA from kernels of the *A. flavus*-resistant peanut germplasm “J11” as a template, gene fragments of *AhSFH8* were cloned via PCR (Fig. 7A). Sequencing results were aligned and analyzed against the corresponding gene sequences in the published genome of cultivated peanut Tifrunner using DNAMAN software. Results demonstrated that the target fragment of *AhSFH8* in resistant material “J11” shared 93.52% identity with that in Tifrunner, with a total length of 1917 bp (Fig. 7B). However, subsequent analysis revealed that translation of this gene terminates at a stop codon located at the 1836 bp position, encoding only 611 amino acids (Fig. 7C). The 1836 bp CDS sequence was employed for all subsequent analysis of *AhSFH8*. To explore the specific role of *AhSFH8* in peanut response to *A. flavus* infection, this study analyzed their expression patterns in kernels of peanut materials with varying resistance levels at different stages post-*A. flavus* inoculation, integrating publicly available transcriptome data from NCBI with RT-qPCR technology. As depicted in Fig. 8, the expression patterns derived from transcriptome data were highly consistent with the RT-qPCR results. At the early stage of *A. flavus* infection (T1), the expression level of *AhSFH8* in the highly resistant material “J11” was slightly lower than that in the highly susceptible material “Zhonghua 12”. With the proliferation of *A. flavus*, by the T2 stage, their expression in “J11” increased sharply, significantly exceeding that in the highly susceptible material, and this high expression state persisted until the T7 stage. These results suggest that *AhSFH8* may play a crucial positive regulatory role during kernel resistance to *A. flavus* infection.

### Overexpression of the *AhSFH8* enhances the resistance of tobacco leaves to *A. flavus* infection

**Fig. 9:**
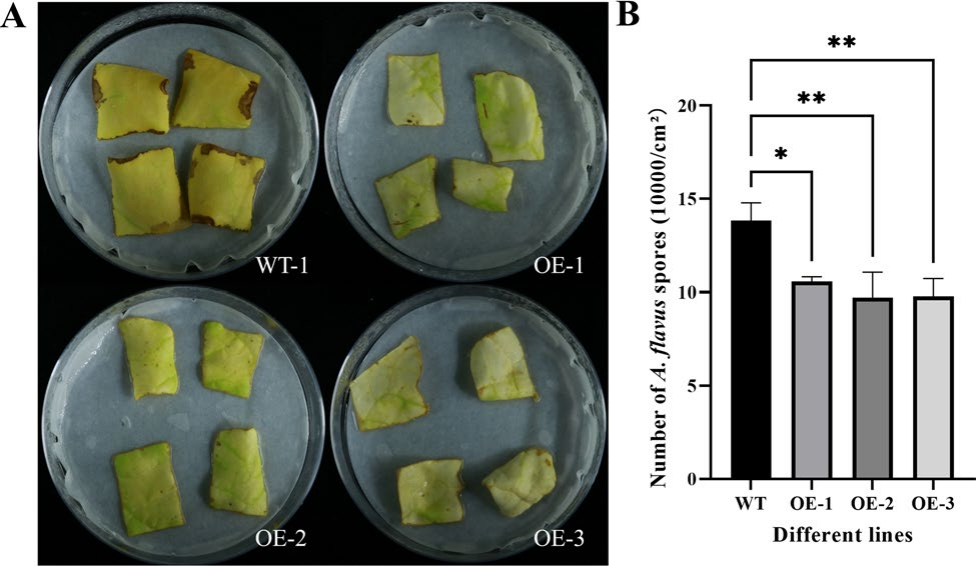
Phenotypic analysis of *AhSFH8* transgenic tobacco responsive to *A. flavus*. **A**, phenotypic differences among leaves of different tobacco lines after inoculation with *A. flavus* spores. **B**, Number of *A. flavus* spores on the leaves surface of different tobacco strains; *: significant at *P* ≤ 0.05. **: significant at *P* ≤ 0.01

Furthermore, the *AhSFH8* gene was introduced into tobacco via the Agrobacterium-mediated leaf disc method, yielding a total of 3 T0 generation overexpressing tobacco plants. Transgenic lines OE-1, OE-2, and OE-3 with uniform and robust growth, along with the wild-type (WT) line, were selected for *A. flavus* inoculation to conduct phenotypic analysis. Results showed that approximately 9 days post-inoculation with *A. flavus*, leaves of WT plants exhibited severe yellowing accompanied by partial lesions, whereas transgenic tobacco leaves displayed mild yellowing without obvious lesions (Fig. 9A). Observations under a 10× light microscope revealed that the spore count in the wild-type line was significantly higher than that in the overexpressing lines (Fig. 9B). These findings indicate that transgenic tobacco exhibits stronger resistance to *A. flavus* compared to wild-type tobacco.

## Discussion

Through genome-wide systematic analysis, a total of 85 members of the PITP gene family were identified in the cultivated peanut (Tifrunner) and classified into three distinct subfamilies: PATL, PITP, and SFH. This classification is consistent with the PITP gene family categorization results reported in other plants, such as *Arabidopsis* and rice [25], indicating that the PITP gene family exhibits high evolutionary conservation. Physicochemical property analysis revealed that parameters including amino acid length, molecular weight, and isoelectric point vary among different members, yet all are predicted to be localized in the cytoplasm. Despite sequence variations among family members, their intracellular localization and biological functions may possess certain degree of conservation. This observation aligns relatively well with the findings of Huang et al. [46], suggesting that these PITPs may play a role in lipid transport and binding, localization, as well as the formation of cellular components. Furthermore, gene structure and conserved motif analysis demonstrated that members within the same subfamily share highly similar intron-exon distribution patterns and conserved motif compositions. This not only further validates the rationality of subfamily classification but also implies that members of the same subfamily may exert analogous biological functions.

Chromosome localization analysis demonstrated that 85 identified *AhPITP* genes are randomly distributed across 20 chromosomes. Among these, 41 pairs of genes were identified as segmentally duplicated, and 1 pair as tandemly duplicated, indicating that segmental duplication is the primary mode of expansion for the peanut PITP gene family, which aligns with the general principle that gene families in plant genomes typically undergo expansion via duplication events [45]. Additionally, a high degree of collinearity was observed between the *PITP* genes of cultivated peanut and those of diploid wild species *A. duranensis*, *A. ipaensis*, as well as other leguminous plants (e.g., alfalfa and soybean). This collinearity suggests that the PITP gene family is relatively conserved during legume evolution and may play crucial roles in their growth, development, and stress responses. As core components of gene transcriptional regulation, promoters determine gene expression patterns and regulatory mechanisms, serving as key entry points for investigating gene expression regulatory networks [47]. Analysis of promoter cis-acting elements revealed that the promoter regions of SFH subfamily members contain a large number of hormone-responsive elements (e.g., ABRE) and stress-responsive elements (e.g., TC-rich repeats), with a proportion significantly higher than that of other subfamilies. This result provides molecular evidence supporting the involvement of SFH subfamily genes in peanut stress responses. Notably, the promoter characteristics of SFH subfamily members in this study are consistent with their high expression patterns following *A. flavus* infection, suggesting that SFH genes may mediate peanut’s response to biotic stress via hormone signal transduction pathways. Furthermore, analysis of FPKM values for SFH subfamily members across different peanut tissues and at various time points after *A. flavus* inoculation showed that most SFH subfamily members have high expression levels in flowers and kernels. At different time points post-*A. flavus* inoculation, *AhSFH8* (*Arahy. HTAW4M.2*), *AhSFH9* (*Arahy. HTAW4M.3*) and *AhSFH10* (*Arahy. HTAW4M.4*), belonging to the SFH subfamily, exhibited significantly higher expression in resistant materials compared to highly susceptible materials, indicating that these genes play important positive regulatory roles in the process of resistant materials combating *A. flavus* infection.

Phosphatidylinositol transfer proteins (PITPs) serve as key regulators at the specific interface between lipid metabolism and phosphatidylinositol signaling in cells [46]. Acting as cellular modulators, they participate in processes such as cell proliferation [48, 49], membrane transport [50, 51], polar growth [52], signal transduction [53], and stress responses [54] through protein-protein or protein-lipid interactions. To preliminarily investigate the specific role of peanut PITP gene *AhSFH8* in resisting *A. flavus*, its coding sequence was successfully cloned from the highly *A. flavus*-resistant material “J11”. The cloned target fragment of *AhSFH8* is 1917 bp in length and it shares 93.52% sequence similarity with the corresponding *AhSFH8* gene in cultivated peanut Tifrunner. Amino acid sequence analysis revealed that *AhSFH8* cloned from “J11” undergoes premature translational termination at 1836 bp. Protein conserved domain analysis showed that it contains the Sec14-like and Nodulin domain-two signature domains of SFH subfamily members, confirming that the cloned *AhSFH8* gene from “J11” encodes a protein belonging to the SFH subfamily of the plant PITP gene family. In previous study, another SFH member, *AhSFH* (*Arahy. E2N38F.1*), was also cloned from “J11” and is hypothesized to play an important positive regulatory role in the early stage of resistant peanut materials defending against *Aspergillus flavus* infection [55]. Interestingly, the first 1836 bp of the *AhSFH8* CDS sequence is completely identical to the full CDS sequence of *AhSFH28* (*Arahy. E2N38F.1*). This observation is speculated to stem from ancient genome duplication events during peanut domestication, which have been retained at a high rate. Expression pattern analysis demonstrated that *AhSFH8* was significantly upregulated in the highly *A. flavus*-resistant material “J11”, with expression peaks peaking at the T2, T3 and T5 stages post-infection, which is consistent with transcriptome data and RT-qPCR verification results [56]. This expression profile suggests that *AhSFH8* may exert its function during the critical periods of peanut resistance to *A. flavus* infection. Tobacco overexpression experiments further validated the anti-*A. flavus* function of *AhSFH8*. Compared with wild-type plants, tobacco leaves overexpressing *AhSFH8* exhibited significantly reduced yellowing after *A. flavus* infection, with spore counts decreasing by over 30%, indicating that this gene can enhance plant resistance to *A. flavus*. This findings align with previous reports that overexpression of sugarcane *ScPITP-1* and *ScPITPp* enhances tobacco resistance to *Ralstonia solanacearum* [21], suggesting that PITP genes possess a conserved function in plant defense against pathogen infection.

Overall, this study represents the first systematic identification of the peanut (*Arachis hypogaea* L.) PITP gene family and clarifies the role of *AhSFH8* in resisting *A. flavus* infection, thereby providing a new perspective for elucidating the molecular mechanisms underlying peanut resistance to *A. flavus*. Nevertheless, this study has certain limitations. First, it has not deeply explored the specific molecular mechanism by which *AhSFH8* regulates peanut resistance *to A. flavus-*for instance, whether it exerts its function through interactions with other proteins or involvement in specific signaling pathways; second, the function of *AhSFH8* has only been verified in a heterologous system (tobacco), and its resistance-related function in peanut requires further confirmation via transgenic peanut experiments. In future studies, we will employ technologies such as yeast two-hybrid assays (Y2H) and RNA sequencing to screen for AhSFH8-interacting proteins and investigate the signaling pathways in which it participates. Additionally, gene editing technology can be utilized to generate AhSFH8 mutants in peanut, aiming to further verify its functional role in peanut resistance to *A. flavus*. These efforts will ultimately provide a theoretical basis and candidate gene resources for molecular breeding of *A. flavus*-resistant peanut varieties.

## Conclusion

This study systematically analyzed the peanut PITP gene family using a combination of genomics, bioinformatics, and molecular biology approaches. A total of 85 PITP gene family members were identified in the genome of cultivated peanut variety Tifrunner, which were classified into three subfamilies (PATL, PITP, and SFH) based on their sequence characteristics. Each subfamily exhibit distinct features in terms of gene structure, conserved motif composition, and chromosomal distribution. Notably, segmental duplication was identified as the primary mode of expansion for the peanut PITP gene family. Analysis of promoter cis-acting elements revealed that members of the SFH subfamily are enriched in hormone-responsive elements and stress-responsive elements, suggesting their important roles in mediating peanut responses to biotic and abiotic stresses. The full-length coding sequence (CDS) of the candidate resistance-related gene *AhSFH8* was cloned from the highly *A. flavus* resistant material “J11”. Expression pattern analysis indicated that within 7 days of *A. flavus* inoculation, *AhSFH8* was significantly upregulated in “J11”, with expression levels peaking at the T2, T3 and T5 stages post-infection, positioning it as a potential candidate gene for peanut resistance to *A. flavus* infection. Tobacco overexpression experiments preliminarily confirmed that the *AhSFH8* significantly enhance resistance to *A. flavus*, as evidenced by reduced leaf yellowing and a more than 30% decrease in spore count on leaves. These results clarify the positive regulatory role of *AhSFH8* in resisting *A. flavus* infection. In summary, this study provides a theoretical basis for elucidating the functions of the peanut PITP gene family and the molecular mechanism underlying peanut resistance to *A. flavus.* Furthermore, *AhSFH8* serve as a candidate gene for the molecular breeding of *A. flavus*-resistant peanuts.

## Supplementary Information

Supplementary Material 1. Fig. S1 Conserved domain analysis of AhPITP proteins. A, Phylogenetic tree; B, Protein conserved domain.

Supplementary Material 2. Fig. S2 Conserved motif sequence of AhPITP proteins.

Supplementary Material 3.

Supplementary Material 4.

## Data Availability

Data presented in this study are available in this research article and supplementary materials.
