# Supplementary figures and images for: Genome-wide identification of the peanut PITP gene family and functional verification of AhSFH8 in resistance to Aspergillus flavus infection
Source: BMC Plant Biol. 2025 Nov 28;25:1661. doi: 10.1186/s12870-025-07667-4 (PMC12664188; doi:10.1186/s12870-025-07667-4)

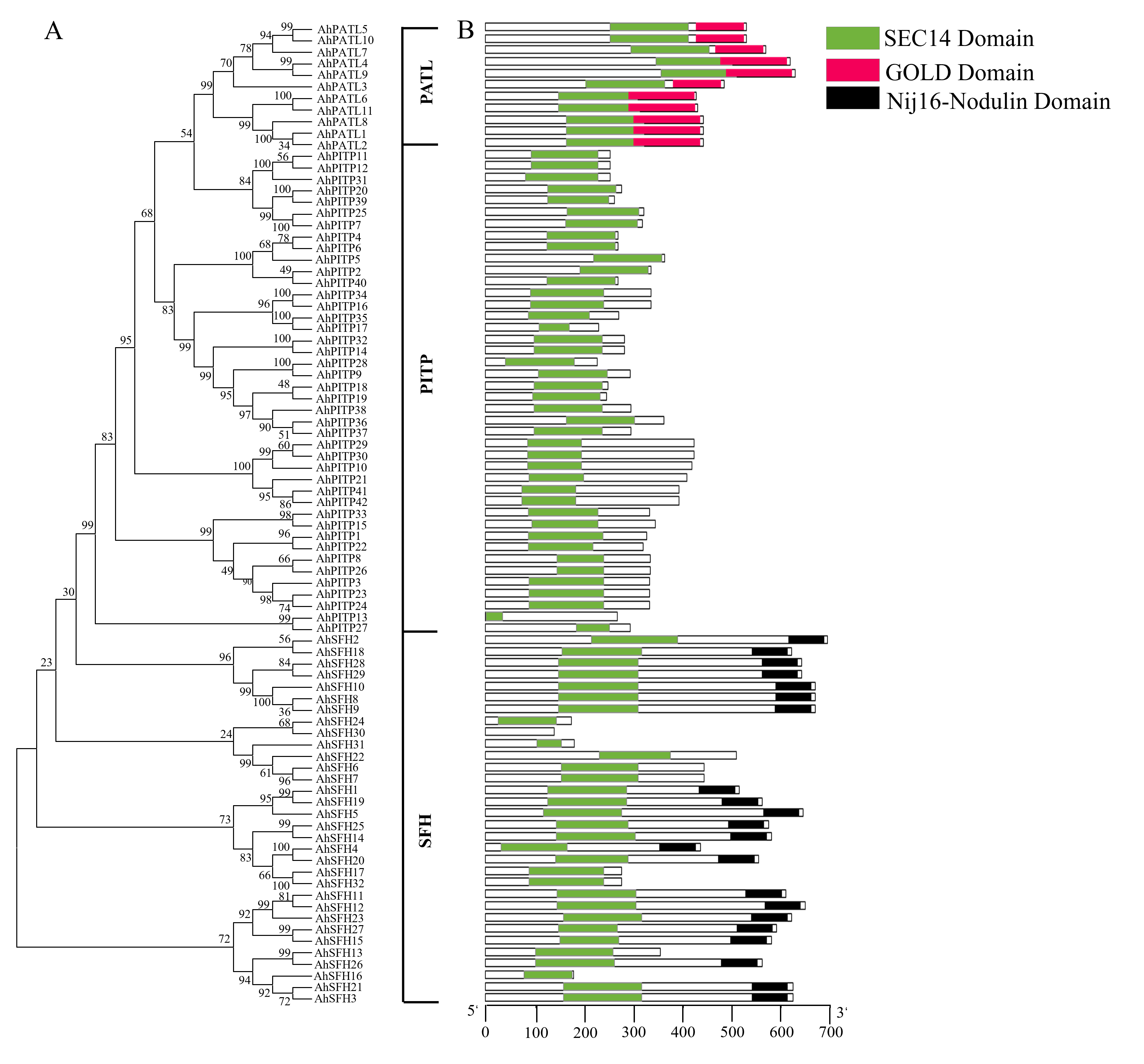

Supplement: Supplementary file 1 — Supplementary Material 1. Fig. S1 Conserved domain analysis of AhPITP proteins. A, Phylogenetic tree; B, Protein conserved domain. [file 12870_2025_7667_MOESM1_ESM.tiff]

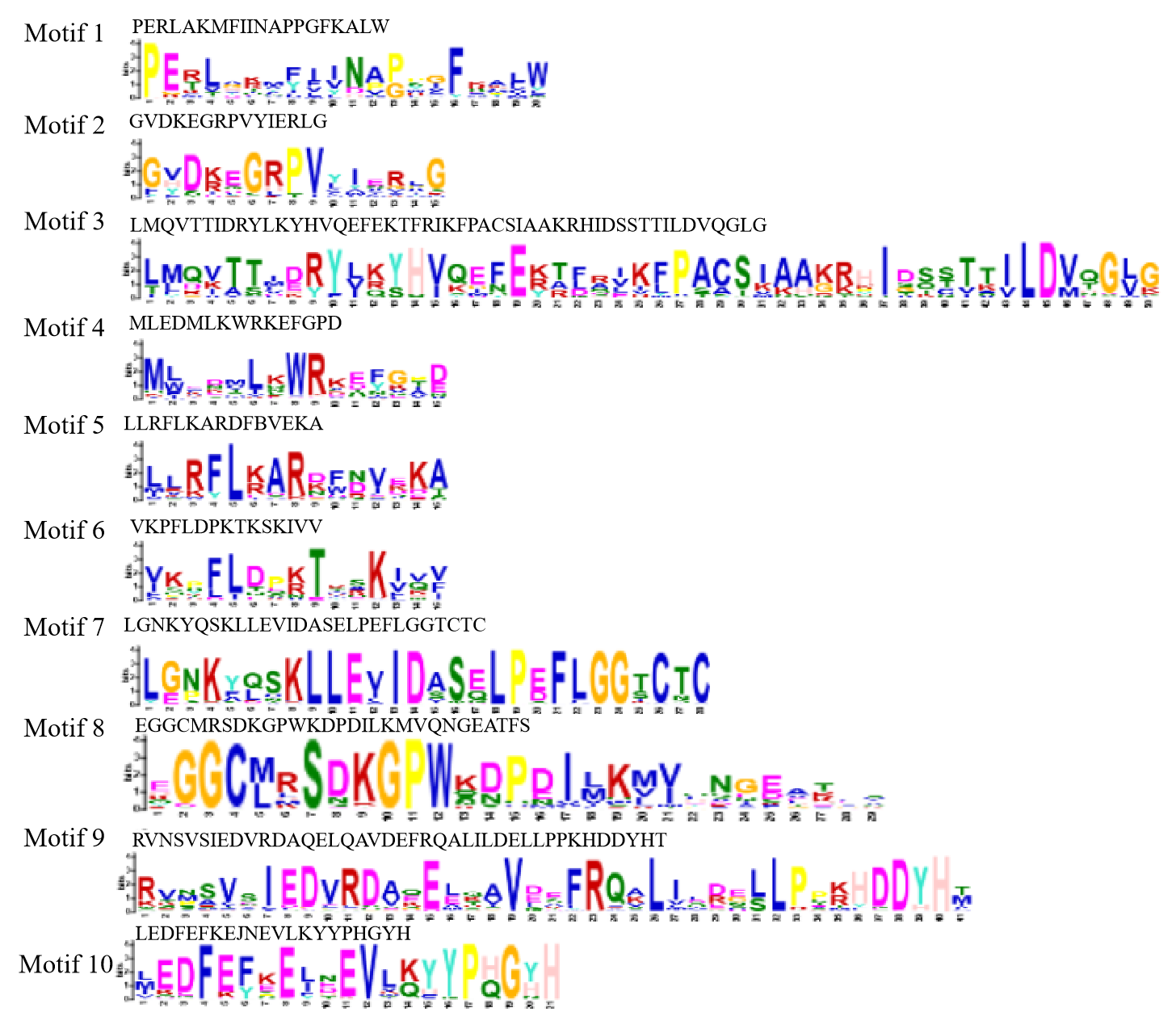

Supplement: Supplementary file 2 — Supplementary Material 2. Fig. S2 Conserved motif sequence of AhPITP proteins. [file 12870_2025_7667_MOESM2_ESM.tiff]

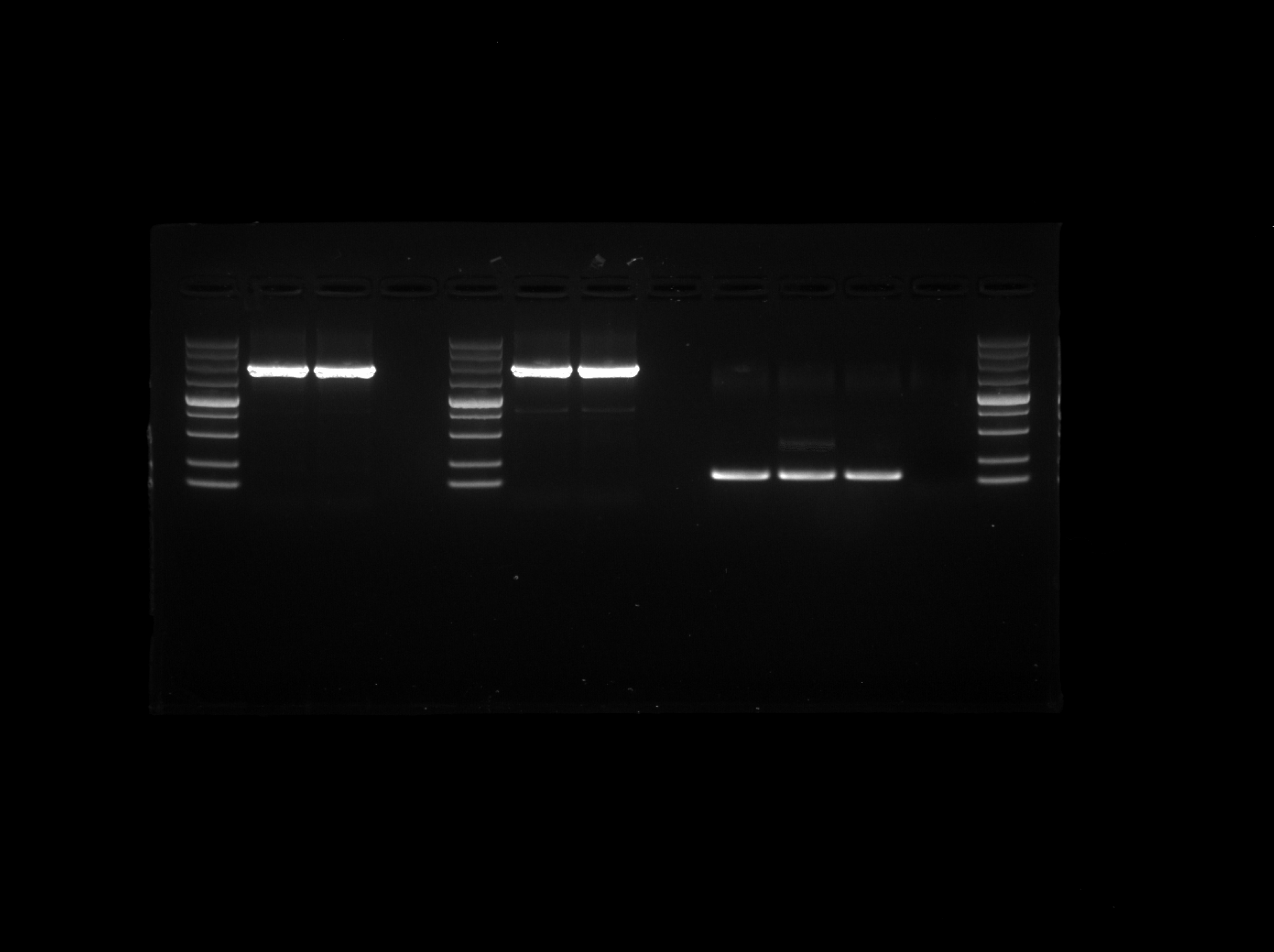

Supplement: Supplementary file 3 — Supplementary Material 3. [file 12870_2025_7667_MOESM3_ESM.zip › Full-Length Original Blots/Blot for Fig.7A.tiff]

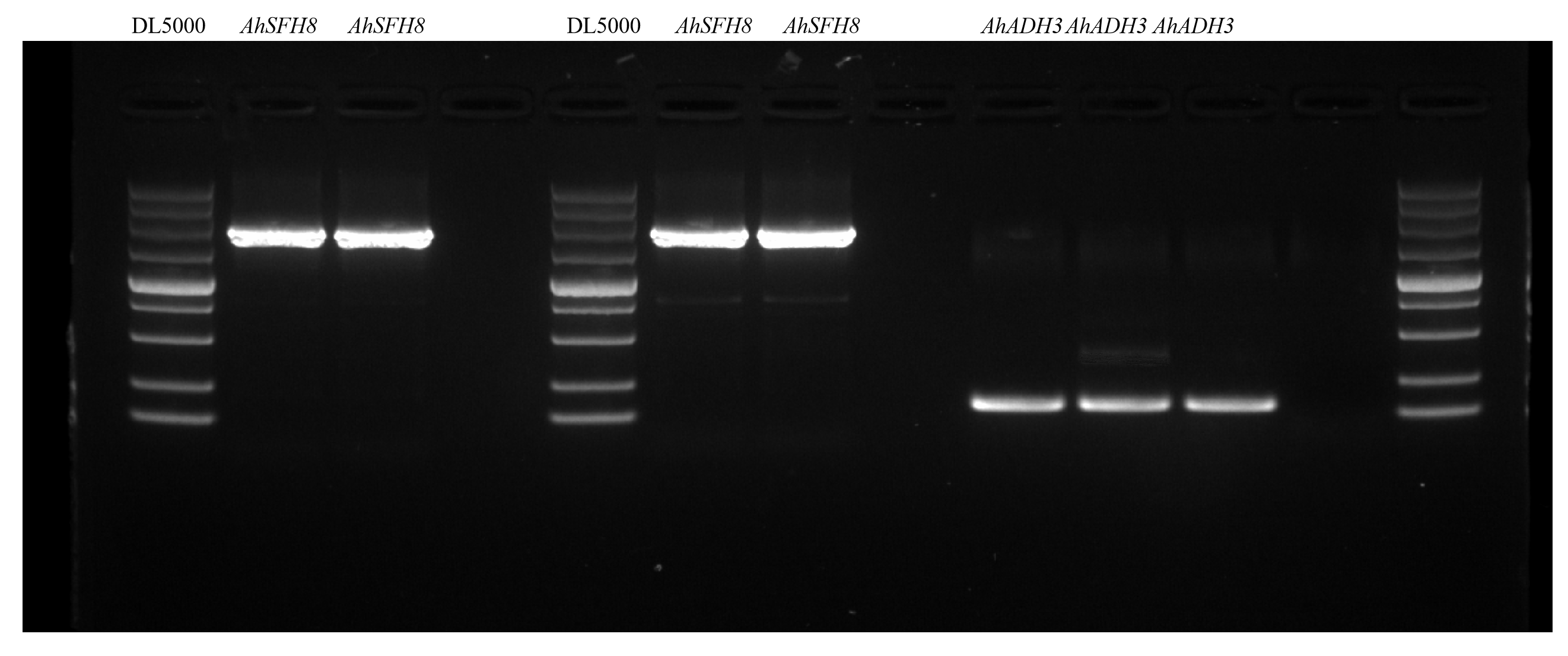

Supplement: Supplementary file 3 — Supplementary Material 3. [file 12870_2025_7667_MOESM3_ESM.zip › Full-Length Original Blots/Descriptors of Fig.7A.tiff]
